# Enhancement in Rate of Photocatalysis Upon Catalyst Recycling

**DOI:** 10.1038/srep35075

**Published:** 2016-10-12

**Authors:** Kalpesh Sorathiya, Biswajit Mishra, Abhishek Kalarikkal, Kasala Prabhakar Reddy, Chinnakonda S. Gopinath, Deepa Khushalani

**Affiliations:** 1 Department of Chemical Sciences, Tata Institute of Fundamental Research, Homi Bhabha Rd, Colaba, Mumbai, 400005 India; 2 Catalysis Division and Center of Excellence on Surface Science, CSIR-National Chemical Laboratory, Pune, 411008 India

## Abstract

Recyclability is an important aspect for heterogeneous photo-catalysts. Ease of recovery and stability of the photo-catalyst in terms of efficiency over the number of cycles are highly desired and in fact it is ideal if the efficiency is constant and it should not decrease marginally with each cycle. Presented here is a seminal observation in which the photocatalytic activity is shown to improve with increasing number of catalytic cycles (it is 1.7 times better after the 1^st^ cycle and 3.1 times better after the 2^nd^ cycle). Specifically, nanorods of pure TiO_2_ and TiO_2_ doped with controlled amount of tungsten have been used to degrade two model pollutants: Phenol and Rhodamine B under exclusive visible light illumination. It was found that, in case of 1 mol.% W incorporation, rate of photocatalysis and also the range of visible light absorption of the photocatalyst increased after the photocatalysis as compared to before photocatalysis. This aspect is unique for doped TiO_2_ and hence provides an intriguing way to mitigate low photoactivity.

Finding easier and more economical ways of activating a diverse set of organic reactions has been the main-stay of a variety of researchers over the years. A catalyst is defined as an additive that lowers the activation energy, providing for a more energetically economical reaction pathway and ideally the catalyst returns to its starting chemical state without any deleterious effects. Photocatalyst is a material that is able to increase the rate of a reaction, however, only when specifically activated by either UV or visible electromagnetic radiation, generating holes and electrons which subsequently participate in oxidation and reduction reactions respectively^1^,^2^,^3^,^4^,^5^,^6^,^7^,^8^.

In terms of the choices of chemical composition for photocatalysis, TiO_2_ has always been the preferred material and used in a wide range of applications (ranging from catalysis,^9^,^10^,^11^,^12^ and as a component in photoanodes in solar cells^13^,^14^,^15^ to even as a scaffold in bone implants^16^). Among the common crystalline forms of titania, anatase is perceived as the more active phase because of its superior surface chemistry, more open framework, and better electron diffusion. This in turn leads to an enhancement in transport efficiency and higher band-gap^17^,^18^,^19^. Furthermore, in order to exploit TiO_2_ even more favorably and to induce improvements in rates of catalysis, (a) its morphology, (b) level of crystallinity and (c) bandgap tuning are some aspects that have been actively manipulated. One morphology, in addition to spherical nanoparticles, that has been oft-cited as being highly conducive for electron transport based applications is 1D nanostructures (nanowires, nanorods and/or nanotubes). These structures can demonstrate faster electron mobility since the direction is confined and they can also display higher sensitivity to the surface processes because of their large surface-to-volume ratio^20^,^21^,^22^,^23^,^24^. Improvement in crystallinity through synthesis has been pursued such that grain boundaries can be minimized (the presence of a large number of interfacial contacts, grain boundaries and a high density of deep electronic states below the conduction band of the TiO_2_ layer slow down the electron diffusion rates due to trapping and detrapping, as a result, these slow electrons are “lost” as their propensity to recombine increases). And finally doping of the chemical structure has been effectively practiced so as to improve the light harvesting capability of TiO_2_. Doping of TiO_2_ with transition metals and non-metals has been extensively studied^25^,^26^,^27^,^28^,^29^,^30^. The variation in absorption properties has been attributed to alteration of the electronic states and interestingly not in all cases does the photoactvity increase. There are many cases where in fact there is a decrease observed. This has been ascribed to the type and concentration of dopant employed and the manner in which it is incorporated in the lattice. Among the cations investigated, Tungsten doping has been showcased quite often due to the fact that the ionic radius is commensurate with Ti^4+^ (W(VI) in a tetrahedral environment has a radius of 56 pm which is the same as Ti(IV) in a tetrahedral environment)^31^,^32^,^33^,^34^. Tungsten with a stable 6 + oxidation state ideally should enable donation up to two extra electrons for every one dopant atom^35^. The ramification of this is that low dopant concentrations are sufficient and in fact are preferred as they lead to reduction in scattering of charge carriers.

Most importantly, an aspect of photocatalysis that normally does not get much recognition is recyclability (especially for heterogeneous catalysis)^36^,^37^,^38^,^39^. In fact, for a catalyst to be industrially viable, it must be able to withstand the reaction conditions repeatedly. The rates of reaction upon re-using the catalysts must not change dramatically (or at all in fact). It is commonly observed that heterogeneous catalysts undergo deactivation or loss in activity mainly due to catalytic poison. In designing a catalyst, ease of recovery and stability in terms of efficiency over the number of cycles are ideally highly desired properties. Normally, literature has emphasized the morphology as a crucial parameter in order to maintain catalytic efficacy over many cycles. Films are preferred as they are easily separated from the solution phase, however nanoparticles offer the highly desirable property of high surface area. Currently, although there are numerous reports on formation of novel photocatalysts and of these very few discuss their recyclability. The latter aspect is only showcased if the rate remains the same or there is a minimal decrease.

As a further to this, presented here is the facile synthesis of tungsten doped TiO_2_ nanorods. The manner of preparation of solely TiO_2_ rods has been adapted from a previous study from our lab where the facile synthesis of an air stable rod-like titanium glycerolate precursor has been developed using glycerol as both a solvent and chelating agent^40^. By calcining at elevated temperature in air, this glycerolate precursor is converted into the anatase phase of TiO_2_ without changing its original morphology. This is a simple environmental-friendly two-step approach without the use of any catalysts, templates, bases or acids. These rods have an aspect ratio that provides for a substantial surface area along with a particle size that provides for ease of recovery from the parent solution. Since the formation of TiO_2_ involves an isolable precursor stage, this methodology lends itself to a unique way of incorporating dopants. Hence we have endeavored to incorporate tungsten at various concentration levels into the precursor itself and then subsequently annealed the precursor to form W doped TiO_2_. Furthermore, these rods have been evaluated for enhanced visible light activity in degrading two separate model pollutants (phenol that is predominantly UV light absorbing and Rhodamine B that is a Visible light absorber) but most intriguingly their photo-catalytic efficiency has been found to be surprisingly improved when the catalyst is recycled.

## Results

**Figure 1:**
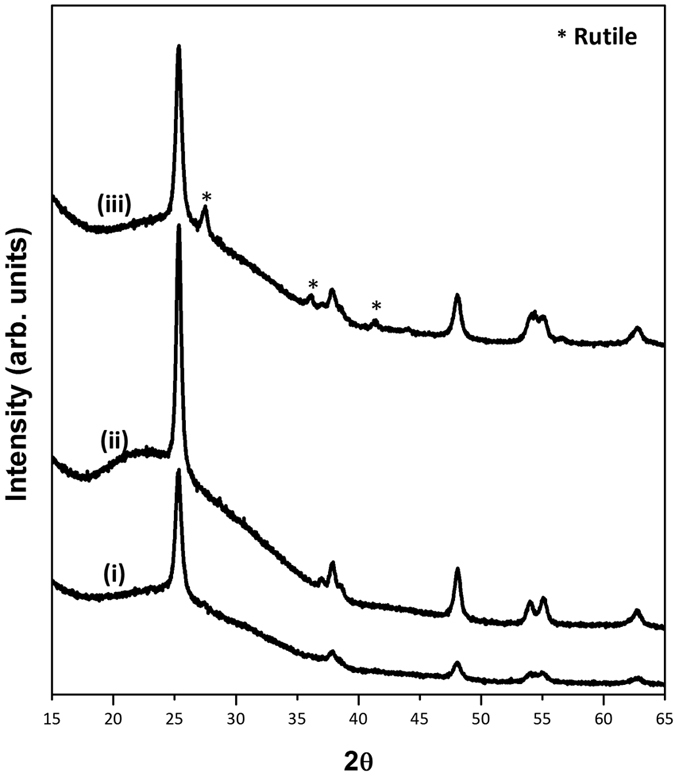
X-Ray diffraction patterns of the as-synthesized photocatalysts (i) TiO_2_,(ii) TiO_2_-1W and (iii) TiO_2_-3W.

**Figure 2:**
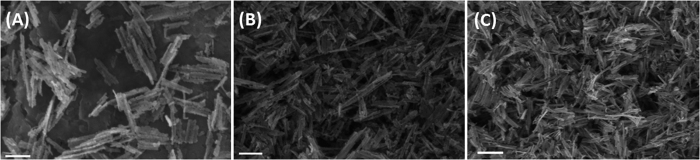
SEM images of (A) TiO_2_,(B) TiO_2_-1W and (C) TiO_2_-3W. Scale bar = 200 nm.

**Figure 3:**
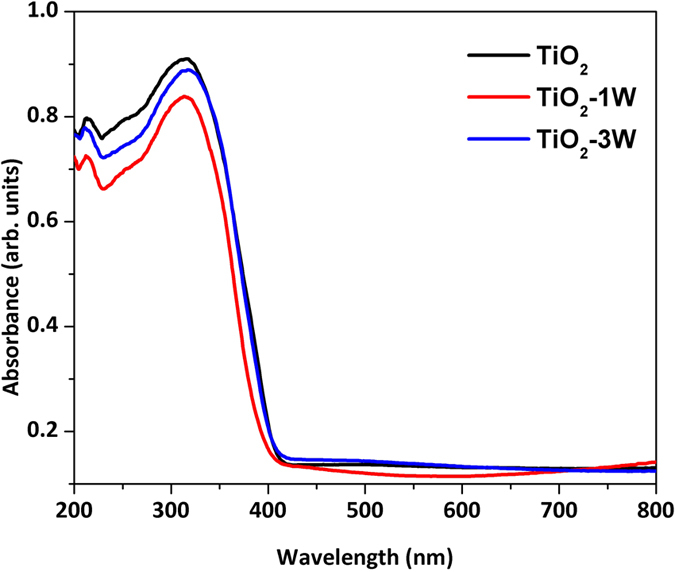
Diffuse reflectance spectra of the three as-synthesized photocatalysts.

For the purposes of this study, 3 samples were prepared. Pure anatase phase of TiO_2_ nanorods, 1 mol.% Tungsten doped TiO_2_ and 3 mol.% Tungsten doped TiO_2_. These have been labeled in the subsequent sections simply as TiO_2_, TiO_2_-1W and TiO_2_-3W respectively. At this point it should be noted that higher loadings of Tungsten were also investigated (namely 5, 7 and 10 mol.%). However as their results were worse than the TiO2-3W, their data is not being detailed here. Room temperature PXRD patterns of the as synthesized TiO_2_ structures are shown in Fig. 1. It can be seen that all the three samples are anatase phase of TiO_2_ (JCPDS 21-1272) however at higher concentration of tungsten loading (3 mol.%) a minor phase of rutile starts appearing. There is absence of any other phases such as WO_3_ in the tungsten loaded samples. In terms of obtaining information regarding the level of crystallinity in the sample, FWHM (full width at half maximum) of two different lattice planes ((101) and (200)) was obtained by fitting a Gaussian curve to these peaks. The data are collated in Table 1 and it can be seen that TiO_2_-1W had the smallest FWHM for both the lattice planes, signifying relatively higher crystallinity than the other two samples. SEM data was collected on all the three samples, Fig. 2, and it can be seen that the morphology consists of rod like particles and this is retained even after incorporating 1 or 3 mol.% of tungsten in the synthesis. Average length of TiO_2_ sample was 220 ± 45 nm, while the average length of rods reduced as the W loading was increased, Table 1. The width of the rods however remained relatively constant. Also shown in Table 1 are the BET surface area values and it can be seen the values were not commensurate with the length of the rods. This is to say, the longest rods (pure anatase) had the BET surface area of *ca*. 61 m^2^/g. However TiO_2_-1W (with shorter rods) also had a smallest BET surface area. Intriguingly, TiO_2_-3W which had the shortest rods had the highest surface area of *ca*. 66 m^2^/g. This part could be attributed to the fact that an additional phase of rutile was observed in this TiO_2_-3W sample, as such this additional phase might not be rod-like and could consist of smaller particles (not resolvable in SEM), hence giving rise to an overall high surface area. Light absorption property of the samples was measured by Diffuse Reflectance Spectroscopy (DRS) which have been shown in Fig. 3. It can be observed that there is no significant change in absorption profile of the three samples after W loading. There is slight blue shift for the TiO_2_-1W sample however there is no subsequent variation for the TiO_2_-3W sample.

### Photocatalysis

**Figure 4:**
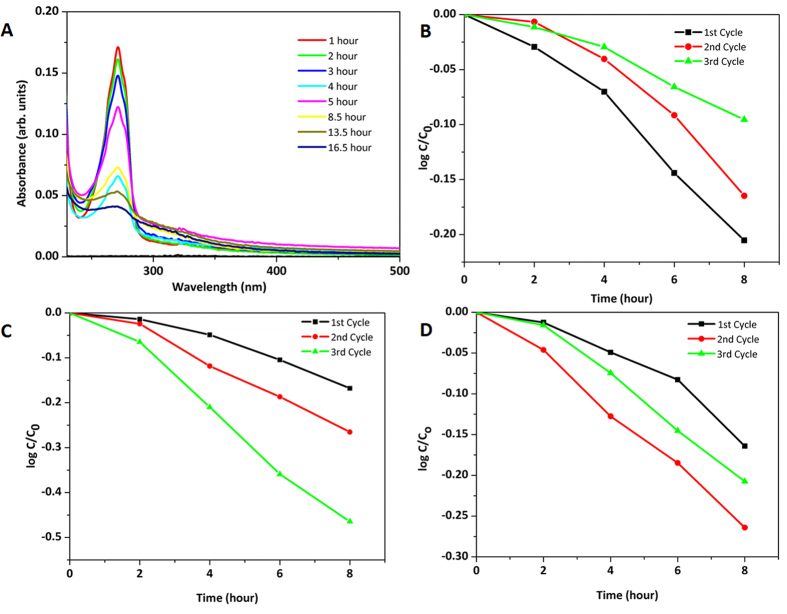
(**A**) Absorption profile of phenol as it undergoes degradation and change in Log C/C_o_ *vs* time for (**B**) TiO_2_ (**C**) TiO_2_-1W and (**D**) TiO_2_-3W.

**Figure 5:**
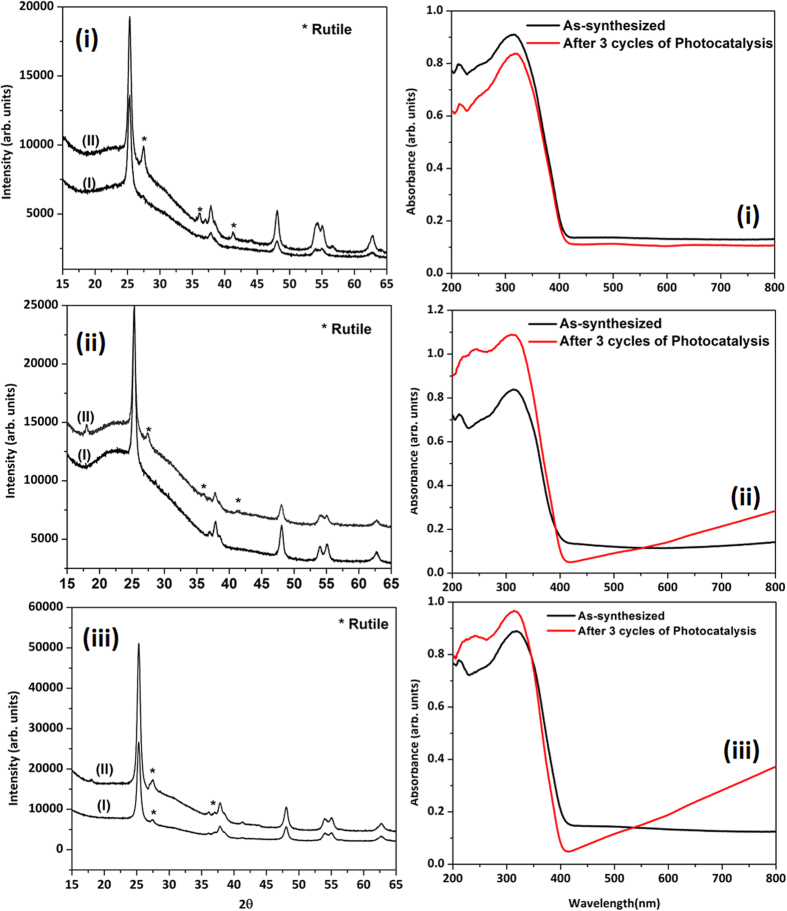
(**A**) XRD of the photocatalysts before (I) and after (II) 3 rounds of photocatlaysis for (i) TiO_2_ (ii) TiO_2_-1W and (iii) TiO_2_-3W and (**B**) Diffuse reflectance spectra of the photocatalysts before and after 3 rounds of photocatlaysis for (i) TiO_2_ (ii) TiO_2_-1W and (iii) TiO_2_-3W.

**Figure 6:**
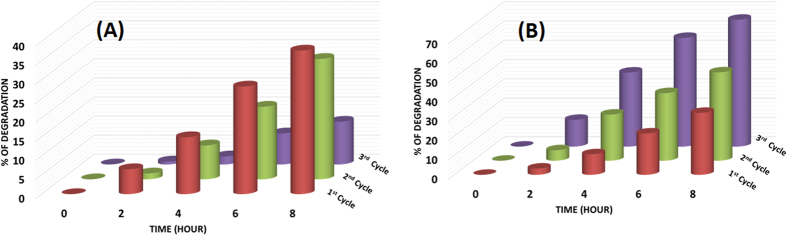
Stack Plot showing % Degradation of Phenol *vs* Time for (A) TiO_2_ and (B) TiO_2_-1W for all the three cycles.

**Figure 7:**
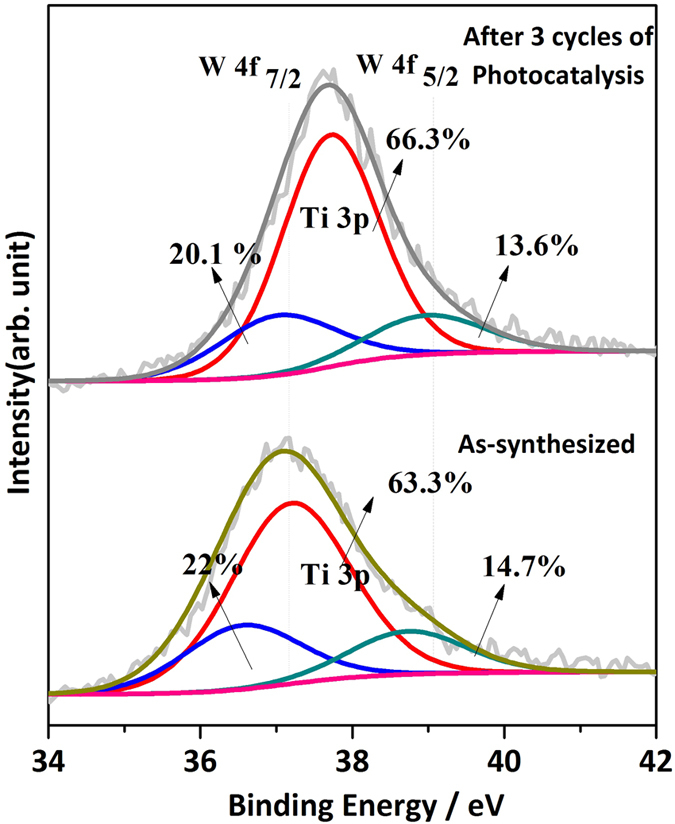
XPS of TiO_2_-1W catalyst before and after 3 rounds of Photocatalysis.

Effectiveness of these samples as photocatalysts was evaluated by performing a degradation experiment using two model pollutants: Rhodamine B and Phenol. For Rhodamine B, excitation of the catalyst was done using only visible light and the data is shown in supplementary information. For Phenol degradation, the samples were also exclusively irradiated with visible light (above 400 nm by using the appropriate long pass filter). Phenol was explicitly used so as to avoid sensitization effects as it has negligible absorption in the visible region hence it is evaluated in detailed here. Figure 4A displays the degradation profile of Phenol with reference to time for TiO_2_. Subsequent to this, recycling of the catalyst was performed whereby, after the 1^st^ round of catalysis, the solid phase was recovered from the phenol solution and the catalyst washed twice with DI water by careful centrifugation at 10,000–14,000 RPM for 10 min. The sample was completely dried and then re-inserted into a fresh solution of phenol and the photocatalysis was redone. Three rounds of such photocatalysis were performed with the same batch of catalyst and in each case a fresh solution of phenol was used. All parameters were controlled as carefully as possible for each cycle (temperature, concentration ratio of catalyst to phenol, volume of solution used, light flux and distance from light source). Figure 4 displays the data as represented by the plot of Log(C/C_0_) *vs.* time for all the three samples and for all the three cycles. For TiO_2_ and TiO_2_-3W sample, it can be seen that the repeated cycling of the same sample leads to an eventual loss in activity and reaction rate, however intriguingly for TiO_2_-1W sample, there is a gradual increase in the rate upon each cycle. The rate constants were obtained using the assumption of a pseudo-1^st^ order reaction rate and the data are compiled in Table 2 (note that the weight of the catalyst has been normalized in all the three rounds of catalysis). The recovered catalysts (after the three cycles were complete) were re-evaluated with XRD, SEM, BET and DRS techniques, and the data are given in Table 2 and Fig. 5. It can be observed that there was a decrease in particle size (*i.e*. rods were getting shorter) after each round of photocatalysis. This was attributed to the stirring of the solid phase leading to a mechanical distress on the morphology for all the three samples. Also observed was that the BET surface area values for all the three samples decreased. The FWHM from XRD data showed that only TiO_2_-1W sample had a slight increase in its peak widths (the other two samples had a decrease in peak widths) suggesting perhaps that the level of crystallinity in these samples was not varying in a similar manner. TiO_2_-1W showed a decrease in crystallinity with each cycle of catalysis. Figure 6 displays a 3D graph that depicts the percent degradation of phenol as a function of time and cycle of photocatalysis. For comparison only TiO_2_ and TiO_2_-1W are shown in this graph and it can be clearly observed that only TiO_2_-1W shows a dramatic increase in the percent of phenol degradation with each cycle. In order to get further insight into the nature of W upon recycling, XPS was performed and the data are shown in Fig. 7. A large peak is observed at 37.1 eV which can be deconvoluted to show the presence of Ti3p and W4f (7/2 and 5/2) peaks. From this, the ratio of Ti to W (before and catalysis) was obtained as 2.9 and 3.3 respectively. Although the ratio does change suggesting a small amount of W leaching during the catalysis, nonetheless it can also be observed that there was negligible change in the binding energy for the main envelope peak and the three deconvoluted peaks for before and after photocatalysis. This insinuated there was no change in the electronic environment of either Ti or W during the three cycles of photocatalysis.

## Discussion

Upon evaluating all the aforementioned data, it can be surmised that only the TiO_2_-1W sample showed dramatic improvement of the photocatalytic activity. The increase in rate of degradation was 1.7 times higher in the second cycle (when compared to the first) and then it was 3.1 times higher in the third cycle (again, when compared to the first). This aspect has been reproduced repeatedly. However this increment was not observed with the other samples. TiO_2_-3W did show a preliminary increase after the first cycle however a subsequent decrease was observed for the latter cycle. Moreover, all these aspects were mirrored in the degradation of Rhodamine B (see supplementary information).

Photocatalytic activity is strongly dependent on parameters such as surface area, band gap, and phase composition (presence of anatase and/or rutile). For all the three samples, after catalysis, a degradation of morphology was observed and the BET surface area seemed to decrease as well. Moreover, in fact out of the three samples, TiO_2_-1W sample had the smallest surface area after the three rounds of catalysis. Despite a decrease in surface area, obviously there was either an increase in the number of charge carriers or charge transport was being enhanced in order to account for the improvement in the catalysis rates. In regards to the phases present after the three cycles of catalysis, TiO_2_ showed a substantial increase in the rutile content, TiO_2_-1W showed a marginal increase while TiO_2_-3W had rutile in the as-synthesized material to begin with and this got augmented after the three cycles of catalysis. Rutile is known to form if the anatase phase is exposed to visible light or is mechanically agitated over prolonged periods^41^,^42^,^43^. Moreover, it is well accepted now in literature that presence of rutile at small concentrations has a beneficial effect on catalysis however at larger concentrations, it has a deleterious effect^17^. These aspects might account for why TiO_2_-3W did not show an enhanced rate in the final cycle. Additionally, normally for tungsten doped TiO_2_, it has been well accepted that two aspects govern the rate of photocatalysis – the surface aspects (presence of W(VI) cation on the surface shifts the isoelectric point to lower values in comparison to pristine TiO_2_) and the bulk aspects (denoted by isomorphic substitution within the lattice)^44^,^45^. It is being hypothesized for this case that as the diffuse reflectance spectrum evolves and there is a slight red shift (*ca*. 0.1 eV) before and after three rounds of catalysis, there is therefore an extension of the light absorption range leading to an increase in the number of charge carriers. Do note that there are no dramatic changes in crystallinity and surprisingly, even the XPS data does not show any change in the oxidation states of W for the TiO_2_-1W sample. The binding energy remains the same before and after the catalysis. Moreover, XRD data shows neither a separated phase of WO_3_ nor other crystalline phases of tungsten. In fact, to improve resolution of the XRD data, long scans (over 12 hours collection time) were done and despite this, additional phases of tungsten oxide were not observed. It can therefore be surmised that non-stoichiometric solid solution of W_X_-Ti_1-X_O_2_ could be feasible because of similarity in ionic radius of the two metal ions. The only slight variation that has been observed, as mentioned above, is the slight red shift of the TiO_2_-1W sample in DRS spectra after catalysis. For the other two samples, the spectra show either a zero or slight blue shift in the absorption profile after catalysis. This suggests that perhaps diffusion (the redistribution of atoms from regions of high concentration, *i.e*. surface, to regions of low concentration, *i.e*. bulk of lattice) is occurring for the dopant cation. It has been reported that when tungsten is the dopant atom, there is a preference for it to reside on the surface of the TiO_2_ nanoparticles. This aspect has been detailed by Couselo *et al.*^45^ and Kong *et al.*^46^. Therefore it is purported for the as-synthesized samples, tungsten which could be residing predominantly only on the surface initially, after each round of photocatalysis, slowly diffuses into the TiO_2_ lattice leading to enhancement. This aspect can be corroborated by the fact that, after catalysis, a slightly higher FWHM is observed only for TiO_2_-1W sample when compared to its two other counterparts. This suggests that there was a decrease in domain size (from which coherent diffraction can occur) upon recycling the catalyst. However for TiO_2_ and TiO_2_-3W, the FWHM showed the opposite trend. This could mean that although the coordination environment around W remains the same, however by having isomorphic substitution into the lattice, the cation dopant is creating shallow states that are leading to enhanced visible light photoactivity with each cycle of photocatalysis (as observed by the increased absorption spectrum range). This aspect is unique in that this study that has revealed a seminal observation that doped catalysts undergo alterations in the nature of their lattice during catalysis. The dopant atom diffuses and impregnates in a more concerted way into the lattice creating a better doped structure leading to enhanced photoactivity. It is important to note that this appears to be only valid for certain concentrations of dopants as at higher concentrations (as in our case for TiO_2_-3W) other effects override the rate of catalysis (such as presence of other phases) that have deleterious effects on the overall rate.

## Conclusion

In summary, TiO_2_ nanowires doped with different concentration of W were synthesized in a simple and facile sol-gel route using glycerol as the solvent and chelating ligand and tungstic acid as the precursor for W. W was incorporated into TiO_2_ lattice as the PXRD pattern did not show the presence of any other phases except TiO_2_. All the photocatalysts were able to degrade phenol and rhodamine B as the model pollutants under exclusive visible light illumination. However, most importantly, it was found that the TiO_2_ nanowires doped with 1 mol.% W showed enhanced photocatalytic activity with increasing number of cycles. This enhancement in photocatalytic activity with increasing number cycles was due to the enhancement in visible light absorption with number of increasing cycles as suggested by the red shift in TiO_2_ absorption band edge in DRS UV-Vis spectra.

## Methods

Analytical reagent grade Titanium butoxide Ti(OCH_2_CH_2_CH_2_CH_3_)_4_ (97%), Anhydrous Glycerol (HOCH_2_CHOHCH_2_OH) and Tungstic acid (H_2_WO_4_) were used without any further purification as received from Sigma Aldrich and Merck respectively. TiO_2_ was prepared by following earlier work reported by Das *et al.*^36^ Primarily, a titanium glycerolate precursor was first synthesized (having a rod like morphology) and this was then subsequently converted to anatase phase of TiO_2_ upon annealing. Specifically, 40 ml glycerol was dried at 140 °C, for preparation of samples having 1% and 3% mole of Tungsten (W) 3.0 mg and 9.0 mg of Tungstic acid powder was added respectively to anhydrous glycerol. Then 0.4 ml Titanium butoxide was added. Reaction mixture was refluxed at 190 °C for 36 Hours under N_2_ flow. A white precipitate of titanium glycerolate was harvested by centrifugation with repeated washings with ethanol. This was dried at 60 °C for 1 hr and then subsequently calcined at 500 °C with a dwell time 1 hour and 1 °C/min heating rate to yield TiO_2_ or its doped analogue.

### Catalyst characterization

Powder X-ray diffraction (XRD) measurements from 5° to 80° 2θ were recorded using PANalytical X’pertpro diffractometer with monochromatic CuKα radiation (λ = 1.54056 Å) operating at 40 kV and 30 mA. BET analysis was done on Micromeritics ASAP Surface Area and Porosity Analyzer. Scanning electron microscopy (SEM) images were taken using JEOL JSM-840. The BET surface area of the sample was measured on Micromeritics ASAP 2020 Analyzer. Prior to N_2_ physisorption data collection, the samples were degassed at 120 °C under vacuum for 12 h. Diffuse reflectance spectra were collected on JASCO V-670 spectrometer. X-ray Photoelectron Spectroscopy (XPS) analysis was performed with a custom built ambient pressure XPS system20^47^ (Prevac, Poland) and equipped with a VG Scienta SAX 100 emission controller monochromator using an Al Kα anode (1486.6 eV) in transmission lens mode. The photoelectrons were energy analyzed using VG Scienta’s R3000 differentially pumped analyzer.

### Measurement of Photocatalytic activity

For measurement of photocatalytic activity, a 100W Schott LED cold source lamp with a 400 nm long pass filter was used to obtain exclusive visible irradiation. 50ml glass beaker was placed having 30 ml of 100 μM phenol solution and 30 mg of catalyst on the magnetic stirrer. Initially in order to attain adsorbed equilibrium, the solution plus catalyst were kept in dark condition for 1 hour with mild stirring. An UV-Vis adsorption reading taken of the phenol supernatant then light was illuminated and after certain time intervals 1.0 ml aliquot was taken for absorbance measurement. Prior to absorbance spectra measurement, the supernatant was centrifuged for 20 mins to remove any catalyst. Absorbance was recorded by SPECORD 205 spectrophotometer from 200–800 nm wavelength range. At the end of each cycle, the catalyst was recovered by centrifugation and air dried. This recovered solid photocatalyst was re-weighed and re-inserted into fresh phenol solution and irradiated with light as the second cycle. Subsequently, again the solid catalyst harvested and reused for 3^rd^ cycle of phenol degradation. Total three consecutive cycles were performed by the same batch catalyst. Volume of phenol solution (1 ml–100 μM) and catalyst (mg) ratio was maintained at 1:1. After the last cycle, the catalyst was calcined to remove any adsorbed impurities and then re-characterized by SEM, DRS, XPS, and BET.

## Additional Information

**How to cite this article**: Sorathiya, K. *et al.* Enhancement in Rate of Photocatalysis Upon Catalyst Recycling. *Sci. Rep.* **6**, 35075; doi: 10.1038/srep35075 (2016).

## Supplementary Material

Supplementary Information

## Figures and Tables

**Table 1:** For the as-synthesized catalysts, the FWHM (from XRD data), average size (from SEM data) and BET surface area values.

Catalyst	FWHM Plane (101)	FWHM Plane (200)	Average Size (L × W nm)	BET Surface Area (m^2^/g)
TiO_2_	0.63	0.66	220 ± 45 **×** 19 ± 2	61 ± 0.4
TiO_2_-1W	0.51	0.58	178 ± 68 **×** 22 ± 6	48 ± 0.1
TiO_2_-3W	0.59	0.64	152 ± 22 **×** 16 ± 4	66 ± 0.2

**Table 2:** Rate constants obtained from each round of catalysis and for the catalysts recovered after 3 cycles of photocatalysis, the FWHM (from XRD data), average size (from SEM data) and BET surface area values.

Catalyst	Rate constants for each cycle of Photocatalysis (hr^−1^)			FWHM Plane (101)	FWHM Plane (200)	Average Size (L × W nm)	BET Surface Area (m^2^/g)
	1^st^ Cycle	2^nd^ Cycle	3^rd^ Cycle				
TiO_2_	−0.024 ± 0.002	−0.017 ± 0.002	−0.011 ± 0.001	0.54	0.61	99 ± 48 **×** 16 ± 2	57 ± 0.2
TiO_2_-1W	−0.018 ± 0.002	−0.031 ± 0.002	−0.057 ± 0.003	0.56	0.60	77 ± 35 **×** 12 ± 3	35 ± 0.1
TiO_2_-3W	−0.017 ± 0.022	−0.030 ± 0.010	−0.023 ± 0.021	0.55	0.60	127 ± 57 **×** 13 ± 6	55 ± 0.1

## References

[b1] Bai S., Jiang J., Zhang Q. & Xiong Y. Steering charge kinetics in photocatalysis: intersection of materials syntheses, characterization techniques and theoretical simulations. Chem. Soc. Rev. 44, 2893–2939 (2015). 25904385 10.1039/c5cs00064e

[b2] Mishra B. & Khushalani D. Nanomaterials based photocatalysts in *Nanocatalysis*: Synthesis and Application (eds Polshettiwar V. & Asefa T.) 469–493 (John Wiley & Sons, 2013).

[b3] Zhang N. *et al.* Waltzing with the versatile platform of graphene to synthesize composite photocatalysts. Chem. Rev. 115, 10307–10377 (2015). 26395240 10.1021/acs.chemrev.5b00267

[b4] Bharad P. A., Sivaranjani K. & Gopinath C. S. A rational approach towards enhancing solar water splitting: a case study of Au–RGO/N-RGO–TiO_2_. Nanoscale. 7, 11206–11215 (2015). 26061862 10.1039/c5nr02613j

[b5] Schneider J. *et al.* Understanding TiO_2_ photocatalysis: mechanisms and materials. Chem. Rev. 114, 9919–9986 (2014). 25234429 10.1021/cr5001892

[b6] Martin D. J. *et al.* Efficient visible driven photocatalyst, silver phosphate: performance, understanding and perspective. Chem. Soc. Rev. 44, 7808–7829 (2015). 26204436 10.1039/c5cs00380f

[b7] Tachikawa T. & Majima T. Single-molecule, single-particle fluorescence imaging of TiO_2_-based photocatalytic reactions. Chem. Soc. Rev. 39, 4802–4819 (2010). 20824247 10.1039/b919698f

[b8] Gligorovski S., Strekowski R., Barbati S. & Vione D. Environmental implications of hydroxyl radicals (•OH). Chem. Rev. 115, 13051–13092 (2015). 26630000 10.1021/cr500310b

[b9] Ohko Y. & Fujishima A. Kinetic analysis of the photocatalytic degradation of gas-phase 2-propanol under mass transport-limited conditions with a TiO_2_ film photocatalyst. J. Phys. Chem. B 102, 1724–1729 (1998).

[b10] Fujishima A. & Honda K. Electrochemical photolysis of water at a semiconductor electrode. Nature, 238, 37–38 (1972). 12635268 10.1038/238037a0

[b11] Graciani J., Nambu A., Evans J., Rodriguez J. A. & Sanz J. F. Au-N synergy and N-doping of metal oxide-based photocatalyst. J. Am. Chem. Soc. 130, 12056–12063 (2008). 18700756 10.1021/ja802861u

[b12] Wilhelm P. & Stephan D. Photodegradation of rhodamine B in aqueous solution via SiO_2_@TiO_2_ nano-spheres. J. Photochem. Photobiol., A: Chemistry 185, 19–25 (2007).

[b13] Roy P., Kim D., Lee K., Spiecker E. & Schmuki P. TiO_2_ nanotubes and their application in dye-sensitized solar cells. Nanoscale 2, 45–59 (2010). 20648363 10.1039/b9nr00131j

[b14] Mishra B., Agarkar S. & Khushalani D. Novel precursors for anatase nanorods and their application in DSSCs. Mater. Chem. Phys. 147, 1110–1116 (2014).

[b15] Mor G. K., Shankar K., Paulose M., Varghese O. K. & Grimes C. A. Use of highly-ordered TiO_2_ nanotube arrays in dye-sensitized solar cells. Nano Lett. 6, 215–218 (2006). 16464037 10.1021/nl052099j

[b16] Gotfredson K., Wennerberg A., Johansson C., Skovgaard C. T. & Hjorting-Hansen E. Anchorage of TiO_2_-blasted, HA-coated, and machined implants: an experimental study with rabbits. J. Biomed. Mater. Res. A 29, 1223–1231 (1995). 10.1002/jbm.820291009 8557724

[b17] Luttrell T. *et al.* Why is anatase a better photocatalyst than rutile? –Model studies on epitaxial TiO_2_ films. Sci. Rep. 4, 4043 (2014). 24509651 10.1038/srep04043 PMC3918909

[b18] Li G. *et al.* Synergistic effect between anatase and rutile TiO_2_ nanoparticles in dye-sensitized solar cells. Dalton Trans. 10078–10085 (2009). 19904436 10.1039/b908686b

[b19] Park N.-G., van de Lagemaat J. & Frank A. J. Comparison of dye-sensitized rutile- and anatase-based TiO_2_ solar cells. J. Phys. Chem. B 104, 8989–8994 (2000).

[b20] Wu W.-Q. *et al.* Hydrothermal fabrication of hierarchically anatase TiO_2_ nanowire arrays on FTO glass for dye-sensitized solar cells. Sci. Rep. 3, 1352 (2013). 23443301 10.1038/srep01352 PMC3583000

[b21] Law M., Greene L. E., Johnson J. C., Saykally R. & Yang P. Nanowire dye-sensitized solar cells. Nat. Mat. 4, 455–459 (2005). 10.1038/nmat1387 15895100

[b22] Liu B., Khare A. & Aydil A. S. Synthesis of single-crystalline anatase nanorods and nanoflakes on transparent conducting substrates. Chem. Commun. 48, 8565–8567 (2012). 10.1039/c2cc33750a 22806181

[b23] Varghese O. K., Paulose M. & Grimes C. A. Long vertically aligned titania nanotubes on transparent conducting oxide for highly efficient solar cells. Nat. Nanotechnol. 4, 592–597 (2009). 19734933 10.1038/nnano.2009.226

[b24] Wang X., He G., Fong H. & Zhu Z. Electron transport and recombination in photoanode of electrospun TiO_2_ nanotubes for dye-sensitized solar cells. J. Phys. Chem. C 117, 1641−1646 (2013).

[b25] Long R. & English N. J. First-principles calculation of nitrogen-tungsten codoping effects on the band structure of anatase-titania. Appl. Phys. Lett. 94, 132102 (2009).

[b26] Serpone N. Is the band gap of pristine TiO_2_ narrowed by anion- and cation-doping of titanium dioxide in second-generation photocatalysts? J. Phys. Chem. B 110, 24287–24293 (2006). 17134177 10.1021/jp065659r

[b27] Gai Y., Li J., Li S.-S., Xia J.-B. & Wei S.-H. Design of narrow-gap TiO_2_: a passivated codoping approach for enhanced photoelectrochemical activity. Phy. Rev. Lett. 102, 036402 (2009). 10.1103/PhysRevLett.102.036402 19257373

[b28] Celik V. & Mete E. Range-separated hybrid exchange-correlation functional analyses of anatase TiO_2_ doped with W, N, S,W/N, or W/S. Phy. Rev. B 86, 205112 (2012).

[b29] Nah Y.-C., Paramasivam I. & Schmuki P. Doped TiO_2_ and TiO_2_ nanotubes: synthesis and applications. Chem. Phys. Chem 11, 2698–2713 (2010). 20648515 10.1002/cphc.201000276

[b30] Kumar A. & Mohanty T. Electro-optic modulation induced enhancement in photocatalytic activity of N-doped TiO_2_ thin films. J. Phys. Chem. C 118, 7130–7138 (2014).

[b31] Abdullah S. M. A. & Chong. F. K. Dual-effects of adsorption and photodegradation of methylene blue by tungsten-loaded titanium dioxide. Chem. Eng. J. 158, 418–425 (2010).

[b32] Song K. Y. *et al.* Preparation of transparent particulate MoO_3_/TiO_2_ and WO_3_/TiO_2_ films and their photocatalytic properties. Chem. Mater. 13, 2349–2355 (2001).

[b33] Grabowska E., Sobczak J. W., Gazda M. & Zaleska A. Surface properties and visible light activity of W-TiO_2_ photocatalysts prepared by surface impregnation and sol–gel method. Appl. Catal. B: Environmental 117–118, 351–359 (2012).

[b34] Pan J. H. & Lee W. I. Preparation of highly ordered cubic mesoporous WO_3_/TiO_2_ films and their photocatalytic properties. Chem. Mater. 18, 847–853 (2006).

[b35] Sathasivam S. *et al.* Tungsten doped TiO_2_ with enhanced photocatalytic and optoelectrical properties via aerosol assisted chemical vapor deposition. Sci. Rep. 5, 10952 (2015). 26042724 10.1038/srep10952 PMC4650706

[b36] Liu Z., Sun D. D., Guo P. & Leckie J. O. One-step fabrication and high photocatalytic activity of porous TiO_2_ hollow aggregates by using a low-temperature hydrothermal method without templates. Chem. Eur. J. 13, 1851–1855 (2007). 17133639 10.1002/chem.200601092

[b37] Roy N., Sohn Y. & Pradhan D. Synergy of low-energy {101} and high-energy {001} TiO2 crystal facets for enhanced photocatalysis. ACS Nano 7, 2532–2540 (2013). 23448713 10.1021/nn305877v

[b38] Han F., Kambala V. S. R., Srinivasan M., Rajarathnam D. & Naidu R. Tailored titanium dioxide photocatalysts for the degradation of organic dyes in wastewater treatment: A review. Appl. Catal. A 359, 25–40 (2009).

[b39] Prado A. G. S., Bolzon L. B., Pedroso C. P., Moura A. O. & Costa L. L. Nb2O5 as efficient and recyclable photocatalyst for indigo carmine degradation. Appl. Catal. A 82, 219–224 (2008).

[b40] Das J., Freitas F. S., Evans I. R., Nogueira A. F. & Khushalani D. A facile nonaqueous route for fabricating titania nanorods and their viability in quasi-solid-state dye-sensitized solar cells. J. Mater. Chem. 20, 4425–4431 (2010).

[b41] Ricci P. C. *et al.* Anatase-to-rutile phase transition in TiO2 nanoparticles irradiated by visible Light. J. Phys. Chem. C 117, 7850–7857 (2013).

[b42] Dutta H., Sahu P., Pradhan S. K. & De M. Microstructure characterization of polymorphic transformed ball-milled anatase TiO2 by rietveld method. Mater. Chem. Phys. 77, 153–164 (2002).

[b43] Pan X., Jiang D., Lin Y. & Ma X. Structural characterization and ferromagnetic behavior of Fe-doped TiO2 powder by high-energy ball milling. J. Magn. Magn. Mater. 305, 388–391 (2006).

[b44] Li X. Z., Li F. B., Yang C. L. & Ge W. K. Photocatalytic activity of WOx-TiO2 under visible light irradiation. J. Photochem. & Photobio. A. 141, 209–217 (2001).

[b45] Couselo N., Einschlag F. S. G., Candal R. J. & Jobbágy M. Tungsten-doped TiO_2_ *vs* pure TiO_2_ photocatalysts: effects on photobleaching kinetics and mechanism. J. Phys. Chem. C 112, 1094–1100 (2008).

[b46] Kong M. *et al.* Tuning the relative concentration ratio of bulk defects to surface defects in TiO2 nanocrystals leads to high photocatalytic efficiency. J. Am. Chem. Soc. 133, 16414–16417 (2011). 21923140 10.1021/ja207826q

[b47] Roy K., Vinod C. P. & Gopinath C. S. Design and performance aspects of a custom built ambient pressure photoelectron spectrometer towards bridging the pressure gap: oxidation of Cu, Ag and Au surfaces at 1 mbar O_2_ pressure. J. Phys. Chem. C 117, 4717–4726 (2013).

