# Supplementary material for: Enhancement in Rate of Photocatalysis Upon Catalyst Recycling
Source: Sci Rep. 2016 Oct 12;6:35075. doi: 10.1038/srep35075 (PMC5059627; doi:10.1038/srep35075)
Supplement: Supplementary Information [file srep35075-s1.doc]

**Supplementary Information**

**Enhancement in Rate of Photocatalysis Upon Catalyst Recycling**

**Kalpesh Sorathiya,^1^ Biswajit Mishra,^1a^ Abhishek Kalarikkal,^1^ Kasala Prabhakar Reddy,^2^ Chinnakonda S. Gopinath,^2^ Deepa Khushalani^1^**^*^

^1^Department of Chemical Sciences, Tata Institute of Fundamental Research, Homi Bhabha Rd, Colaba, Mumbai, India 400005

^1a^Current Address: CSIR- Institute of Minerals and Materials Technology, Bhubaneswar, Odisha, India 751013

^2^Catalysis Division and Center of Excellence on Surface Science, CSIR-National Chemical Laboratory, Pune, India 411008

[*Khushalani@tifr.res.in](mailto:*Khushalani@tifr.res.in)
